# Direct Analysis of Low-Volatile Molecular Marker Extract from Airborne Particulate Matter Using Sensitivity Correction Method

**DOI:** 10.1155/2016/2834575

**Published:** 2016-04-04

**Authors:** Satoshi Irei

**Affiliations:** Centre for Atmospheric Chemistry and Department of Chemistry, York University, 4700 Keels Street, Toronto, ON, Canada M3J 1P3

## Abstract

Molecular marker analysis of environmental samples often requires time consuming preseparation steps. Here, analysis of low-volatile nonpolar molecular markers (5-6 ring polycyclic aromatic hydrocarbons or PAHs, hopanoids, and* n*-alkanes) without the preseparation procedure is presented. Analysis of artificial sample extracts was directly conducted by gas chromatography-mass spectrometry (GC-MS). After every sample injection, a standard mixture was also analyzed to make a correction on the variation of instrumental sensitivity caused by the unfavorable matrix contained in the extract. The method was further validated for the PAHs using the NIST standard reference materials (SRMs) and then applied to airborne particulate matter samples. Tests with the SRMs showed that overall our methodology was validated with the uncertainty of ~30%. The measurement results of airborne particulate matter (PM) filter samples showed a strong correlation between the PAHs, implying the contributions from the same emission source. Analysis of size-segregated PM filter samples showed that their size distributions were found to be in the PM smaller than 0.4 *μ*m aerodynamic diameter. The observations were consistent with our expectation of their possible sources. Thus, the method was found to be useful for molecular marker studies.

## 1. Introduction

Studying chemical composition of airborne particulate matter (PM) is very important to better understand the radiative forcing [1] and the adverse health effect [2]. Organic fraction in airborne PM (referred to as particulate organic matter or POM hereafter) is potentially associated with these issues. There is no doubt that the airborne POM is the major constituent of airborne PM [3]. To date, numerous field studies have been done for identification of major primary POM sources and their source apportionment [4–7]. Those studies often use fingerprinting organic substances, called molecular markers. Trace level molecular marker analysis is tedious and time consuming. The analysis usually involves 4 major steps: extraction, preseparation, concentration, and measurement. A preseparation step using solid-phase extraction (SPE) technique provides more reliable quantitative analysis for trace level molecular markers in extracts with complex compositions. However, the step requires additional pricy consumables (~20% of whole cost for purchasing consumables) and substantial amount of processing time (maybe ~30% of whole analysis time). If successful analysis was made without this procedure, it would save considerable amount of budget, time, and labor.

The objective here is to establish a simple measurement method for selected nonpolar molecular markers (listed in the following section) using a technique of gas chromatography-mass spectrometry (GC-MS): the direct analysis of POM extracts without a usual preseparation step using column or cartridge chromatography. Qualitative and quantitative analysis were made by combination of scan monitoring and selected ion monitoring (SIM) of MS. Sensitivity deterioration due to the matrix effect was corrected based on the sensitivity profile made by injecting a standard mixture after an injection of sample extract. Standard-spiked tests and the analysis of NIST standard reference material (SRM) 1975 diesel particulate matter extract and SRM 1650 diesel particulate matter were performed to validate the methodology. Additionally, the methodology was further validated by analyzing ambient PM samples collected during the Southern Ontario aerosol study. Brief results of molecular marker concentrations using this method will be presented.

## 2. Experiment

The following low-volatile nonpolar molecular markers were targeted for quantitative analysis: five PAHs of benzo[*a*]pyrene (BaP), dibenz[*a*, *h*]anthracene (Db), indeno[*1,2,3-cd*]pyrene (Ind), benzo[*ghi*]perylene (BgP), and coronene (Cor); four hopanes of trisnorhopane (TrisHp), norneohopane (NorHp), *α*, *β*-hopane (abHp), and *β*, *α*-hopane (baHp); and 15* n*-alkanes from C_20_ to C_34_. A 16-PAH standard mixture that contained 2000 ± 10 *μ*g mL^−1^ of each PAH in dichloromethane/benzene mixture (>99%, Ultra Scientific, USA) was used as the primary standard mixture for BaP, Db, Ind, and BgP. The primary standard solution of coronene was prepared by dissolving the pure reagent (>99%, Sigma-Aldrich Canada, Oakville, Canada) in HPLC grade hexane (Sigma-Aldrich Canada). The primary standard mixture of hopanes was prepared from the commercially available 100 ± 5 *μ*g mL^−1^ hopane standard solutions in isooctane (Chiron AS, Trondheim, Norway). The* n*-alkane primary standard mixture was prepared by dissolving the pure reagents of C_20_ to C_34_ *n*-alkanes (>99%, Sigma-Aldrich Canada) in hexane (PRA grade, Sigma-Aldrich Canada). Those primary standard solutions were used for the calibration, the standard-spiked tests, and the standard for checking the instrumental sensitivity routinely. In addition, benzo[*a*]pyrene-*d*_2_ (BaP*d*_2_) was used as internal standards for quantitative analysis of the PAHs, and tetracosane-*d*_50_ (C_24_ *d*_50_) (Sigma-Aldrich Canada) was used for the analysis of the hopanes and* n*-alkanes in the standard-spiked test. To calculate the recovery yields in the standard-spiked test, an internal standard for recovery control (ISRC), triacontane-*d*_62_ (C_30_ *d*_62_), was spiked to the final extracts. For ambient sample analysis, this C_30_ *d*_62_ was also used as the usual internal standard together with the BaP*d*_2_ and the C_24_ *d*_50_. The following is a description of the analytical procedure for filter samples.

**Figure 1:**
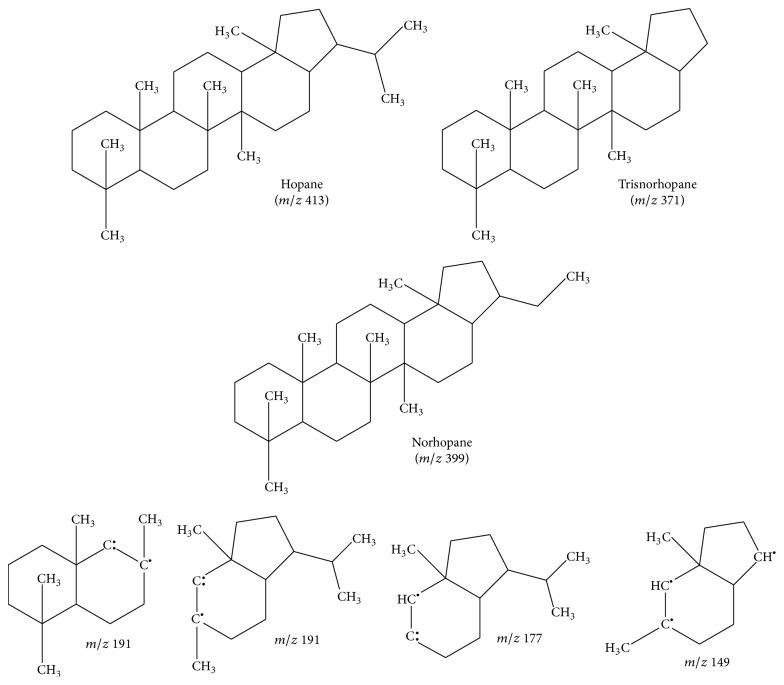
Molecular structures of targeted hopanoid series and possible structures of their fragment ions.

A quarter piece of an 8 × 10 inch quartz fiber filter (Tissuequartz 2500 QAT-UP, Pall Corp., NY, USA) was spiked with the internal standards and extracted at 313 K for 16 hours with 100 mL of PRA grade dichloromethane (Sigma-Aldrich Canada, Oakville, ON, Canada) using Soxhlet apparatus (Sigma-Aldrich Canada). Volume of the extract was reduced to a few mL using a rotary evaporator (Büchi, New Castle, DE, USA). The concentrated extract was then filtered using a 2 mL gas-tight syringe (Hamilton, Reno, NV, USA) with a 0.45 mm PTFE syringe filter (Chromatographic Specialties, Inc., Brockville, ON, Canada) and transferred into a 5 mL Reacti-Vial (Pierce, IL, USA). The volume of the filtered extract was further reduced to ~0.2 mL under a gentle stream of pure nitrogen (Praxair Canada, Mississauga, Canada). A 5 *μ*L of the concentrated extract was then directly analyzed by a gas chromatography-mass spectrometry or GC-MS (HP 5890 and 5972, Agilent Technology, USA). A surface-deactivated injection sleeve and glass wool (Siltek split/splitless sleeve and Siltek glass wool, Restek Corp., Bellefonte, USA) were used for the analysis. 5Sil-MS (0.25 mm i.d. × 30 m with 0.25 mm film thickness, Restek Corp.) was used as the separation column. At the injection, splitless injection mode was held for 1 min. Optimized GC temperature program for separation was as follows: the initial isothermal hold at 373 K for 0.1 min, then ramping temperature at rate of 5 K min^−1^ to 398 K with its isothermal hold for 3 min, and then ramping temperature at 5 K min^−1^ to 573 K with its isothermal hold for 8 min. Temperature for the injector and the interface between the GC and the MS was set to 573 K during the analysis. Flow rate of carrier gas, helium, was set to 1 mL min^−1^ continuously. Combination of scanning and selected ion monitoring (SIM) modes was used to identify and quantify the molecular markers. Mass-to-charge ratios (*m/z*) of the designated ions were chosen in a way that the molecular and fragment ions were unique (except those for* n*-alkanes) and the major ions in the mass spectra obtained by the standard analysis. The selected ions are listed in Table 1. It was found that the most of selected ions for PAHs were molecular ions (M^+^ and M^2+^ ions). For* n*-alkanes, the selected ions were C_6_H_13_^+^ and C_7_H_15_^+^. For the hopane series, the structures of parent molecules and fragment ions selected are shown in Figure 1. The structures of the fragment ions were based on Soares et al. [8]. The identification of the molecular markers was made by comparison with the retention time and the reference mass spectrum obtained by analysis of the chemical standards, as well as with NIST 98 mass spectrum library. As quality assurance, a reference standard mixture containing 100 ng mL^−1^ of each molecular marker referred to earlier was measured after the analysis of one sample extract.

Three types of measurement tests were carried out to validate the analytical methodology: recovery yield tests using spiked filters with a reference standard mixture; direct measurements of NIST SRM 1975 diesel particulate matter extract; and analysis of NIST SRM 1650 diesel particulate matter. For the standard-spiked tests, specific volume of the standard solutions was spiked to a prebaked quartz fiber filter, followed by the solvent extraction. For the experiment with SRM 1650, the powder of SRM was spread on a preweighted filter using a small spatula and the mass of SRM on the filter was weighted using an ultra-micro balance (Mettler Toledo LLC, Columbus, USA).

8 × 10 inch quartz fiber filters previously referred to were used for collection of PM. Prior to the sampling, all filters were baked at 1023 K for at least 4 hours in a muffle furnace (Model 550-58, Fisher Scientific) that was flushed with dry synthetic air (Matheson Gas Products) during the baking. The baked filters were stored in thoroughly precleaned glass dishes with 0.1 M nitric acid (BDH), acetone (HPLC grade, BDH), dichloromethane (PRA grade, Sigma-Aldrich Canada), and Milli-Q water (Millipore Corp., Billerica, USA). Those filters were stored in clean deep freezers under 253 K until used for the sampling.

The Southern Ontario aerosol study 2000 (SONTAS 2000) was conducted at Hamilton and Simcoe, which represent an industrial and a rural site in Southern Ontario, respectively. Tunnel studies were also conducted at the York Gateway tunnel in downtown Toronto to compare the results from the field study aforementioned with the molecular markers from vehicular emissions. High-volume air samplers with a PM_10_ separator head (TE-6070BL, Tish Environment, Inc., Village of Cleves, USA) or the separator head together with a 6-stage (10 *μ*m < aerodynamic diameter (*D*_*p*_) < 7.2 *μ*m; 7.2 *μ*m < *D*_*p*_ < 3.0 *μ*m; 3.0 *μ*m < *D*_*p*_ < 1.5 *μ*m; 1.5 *μ*m < *D*_*p*_ < 0.95 *μ*m; 0.95 *μ*m < *D*_*p*_ < 0.49 *μ*m; and *D*_*p*_ < 0.49 *μ*m) cascade impactor (Series 230, Tish Environment, Inc.) were used for the PM sampling. Flow rates of the samplers were calibrated with a manometer at the beginning of the studies. The sampling flow rates were 1.13 m^3 ^min^−1^. Sampling duration during the SONATS 2000 and the tunnel studies was 24 hours and 4 hours, respectively. These sampling periods corresponded to a collection of air volume of 1627 m^3^ and 271 m^3^, respectively. Collected filter samples were stored in the clean jars previously referred to under 253 K until analyzed.

## 3. Results and Discussion

### 3.1. Detection Limit, Blank Value, and Sensitivity Correction

Reproducibility of GC-MS analysis in SIM mode for the targeted compounds was typically better than 10%, and instrumental and atmospheric detection limits (DLs) and field blank values are listed in Table 2. An instrumental DL is defined as three times the standard deviation (*σ*) or 3*σ* of peak area counts integrated at the retention time for a targeted compound peak in chromatograms obtained from multiple blank GC runs (*n* > 3). A field blank value for a targeted compound is defined as its average concentration obtained from the analysis of field blank filters (*n* = 16), which underwent all the handling procedures that a real filter sample undergoes, including the sample transport and storage, except for air sampling. An atmospheric DL for a targeted compound is defined as the quotient of 3*σ* of its field blank values divided by 1627 m^3^, a typical air volume sampled by a high-volume air sampler for filter sampling. It should be noted that the field blank and the atmospheric DLs discussed hereafter are given as per a whole filter basis. It was found that the magnitudes of the field blank values and the atmospheric DLs for the PAHs and the hopanes were less than 2% and 4% of observed mean concentration at Simcoe, respectively. These values were low enough to determine their atmospheric concentrations. However, for the* n*-alkanes the blank values were 4%–60% of the observed concentrations at Simcoe, and no systematic trend on the carbon number was observed. The large variations were suspected to be due to irregular contamination from ubiquitous wax containing those* n*-alkanes.

**Figure 2:**
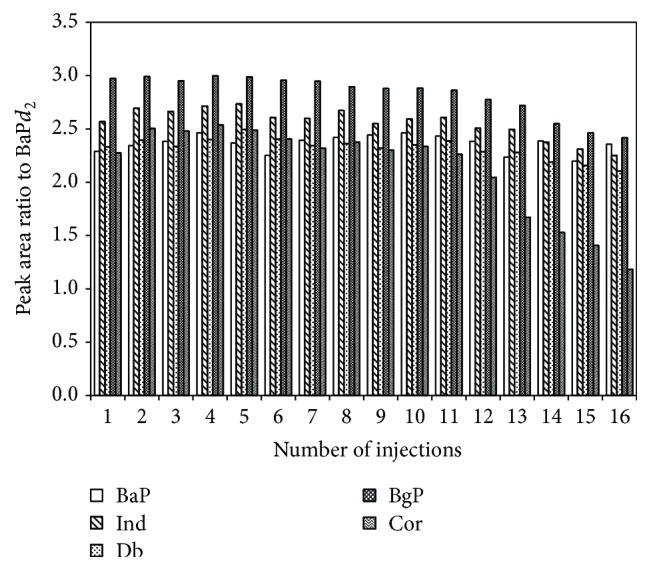
Variation of peak area ratios of targeted PAHs (100 ng injection) relative to the peak area of internal standard (BaP*d*_2_) as a function of injection number.

It was found that the more injections of the sample extracts, the more deterioration of peak area counts. The routine injection of the molecular marker mixture after the injection of the sample extract exhibited the sensitivity deterioration (Figure 2). Such sensitivity deterioration was observed only for the PAHs. This deterioration was due to the cross-contamination from the previous injections; residuals of unfavorable matrix in the inlet resulted in inefficient transfer of the PAHs into the GC column. The sensitivity was restored by replacing the GC inlet liner and cutting the tip of GC column. Such maintenance was made after 10–15 measurements of the sample extracts. If necessary, corrections on peak area counts were made using the sensitivity profile shown in Figure 2.

### 3.2. Standard-Spiked Test

Table 3 shows the spiked masses, the determined masses based on the internal standard method, the recovery yields determined by a use of the ISRC, and the recovery yield ratios to the recovery yield of the internal standard (BaP*d*_2_ for the PAHs and C_24_ *d*_50_ for the hopanes and the* n*-alkanes). The recovery yields of the hopane series were 74–90%, while those of the PAHs and the* n*-alkanes were 44–64% and 78–133%, respectively. The low recovery yields of the PAHs are probably attributed to their losses during the extract preparation procedure. Because the PAHs are slightly more polar than the hopanes and* n*-alkanes, this weak polarity may have caused the compounds to adhere to the glass wall of experimental apparatuses. Meanwhile, the recovery yields of* n*-alkanes deviated largely. This problem is probably due to the difference in retention time between the C_24_ *d*_50_ internal standard and other* n*-alkanes. Although the recovery yields of the internal standards were not perfectly identical to those of the target compounds, the recovery yield ratios were reproducible; thus, it was concluded that the use of the internal standards for the analysis of these molecular markers was acceptable.

### 3.3. SRMs

Reference values of SRM 1975 diesel particulate matter extract were given for BgP and Ind and those of 1650 diesel particulate matter were given for BaP, Ind, and BgP by NIST. Thus, measured values were compared only for these compounds (Tables 4 and 5). The measurement results of SRM 1975 demonstrated that the measurement values of BgP and Ind deviated, but the average values agreed with the reference values within the range of standard deviation (16-17%). This indicates that the interference of another matrix in the extract was insignificant.

For SRM 1650, the reference values have been issued several times (1650 in 1988 [9], 1650a in 1999 [10], and 1650b in 2013 [11]) due to the update of the PAH concentrations. Our measurements for SRM 1650 demonstrated larger deviations (31–34%) than the measured values for SRM 1975. The larger standard deviations are likely attributed to the uncertainties of the mass measurements of SRM 1650 in microgram order as well as the extraction efficiency. It has been discussed that, compared with accelerated solvent extracting method, Soxhlet, our extraction method, was prone to result in lower concentration for high molecular weight PAHs, such as Ind and BgP [12]. Our measurements for BaP gave the average value within the standard error of the mean (1*σ*), while Db, Ind, and BgP gave the average values in between the reference values of 1650 and 1650b, showing small biases. The average values still agreed with the most recent reference values within the 2*σ* range. Overall, the analysis test with SRM 1650 validated our PAH measurements with the uncertainty of 30% approximately.

### 3.4. Analysis of PM Filter Sample Extracts

**Figure 3:**
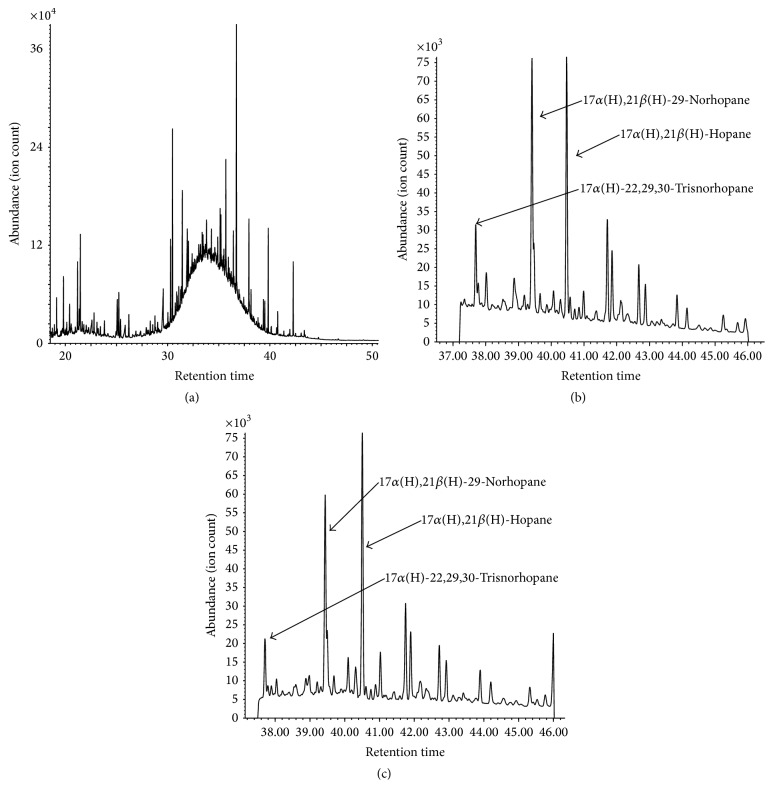
(a) Total ion chromatogram by Scan for the July 13 sample at Simcoe; (b)* m/z* 191 ion chromatogram by SIM for June 1 at York Gateway tunnel; (c)* m/z* 191 ion chromatogram by SIM for June 23 at Hamilton.

**Figure 4:**
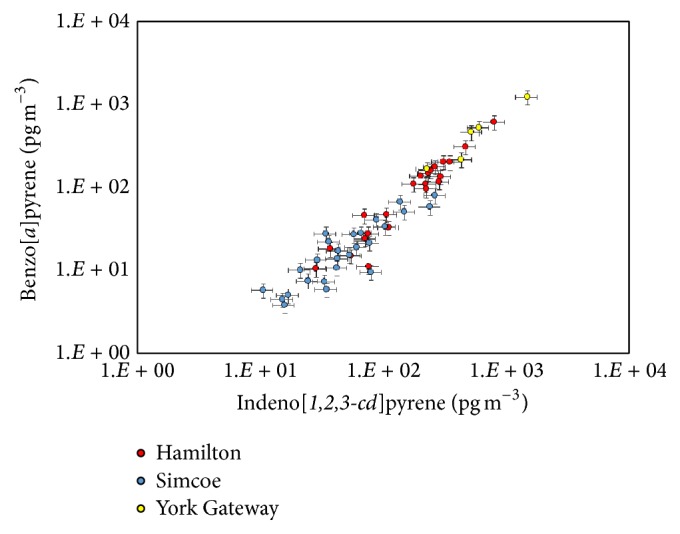
Log-log plot for benzo[*a*]pyrene concentration as a function of indeno[1,2,3-* cd*]pyrene concentration observed at York Gateway tunnel (yellow), Hamilton (red), and Simcoe (light blue).

The analysis of ambient sample extracts demonstrated the usefulness of the methodology for the molecular marker analysis. Many chromatograms obtained from the analysis of filter samples collected at Hamilton and some from those collected at Simcoe showed a hump attributing to an unresolved complex mixture or UCM (Figure 3(a)). The hump is an indication of organics from fossil fuel combustion [13]. Indeed, the* m/z* 191 ion chromatograms for hopanes, molecular markers for fossil fuel combustion (Figure 3(b)), also showed detection of those marker substances (Figure 3(c)). In addition, the PAH analysis also demonstrated that the BaP concentrations observed at the two locations in the southern Ontario and the York Gateway tunnel were highly correlated with the Ind concentrations (Figure 4). The coefficient of determination for the linear regression from all the data points was 0.997. Such high correlations were observed between the molecular markers in the same family only (i.e., between the PAHs, hopanoids, or* n*-alkanes), probably due to different production processes [14]. This high correlation suggests that these PAHs can be used as source identification of vehicular emissions. Even though the plot is not shown, high correlation was observed between hopane series as well.

**Figure 5:**
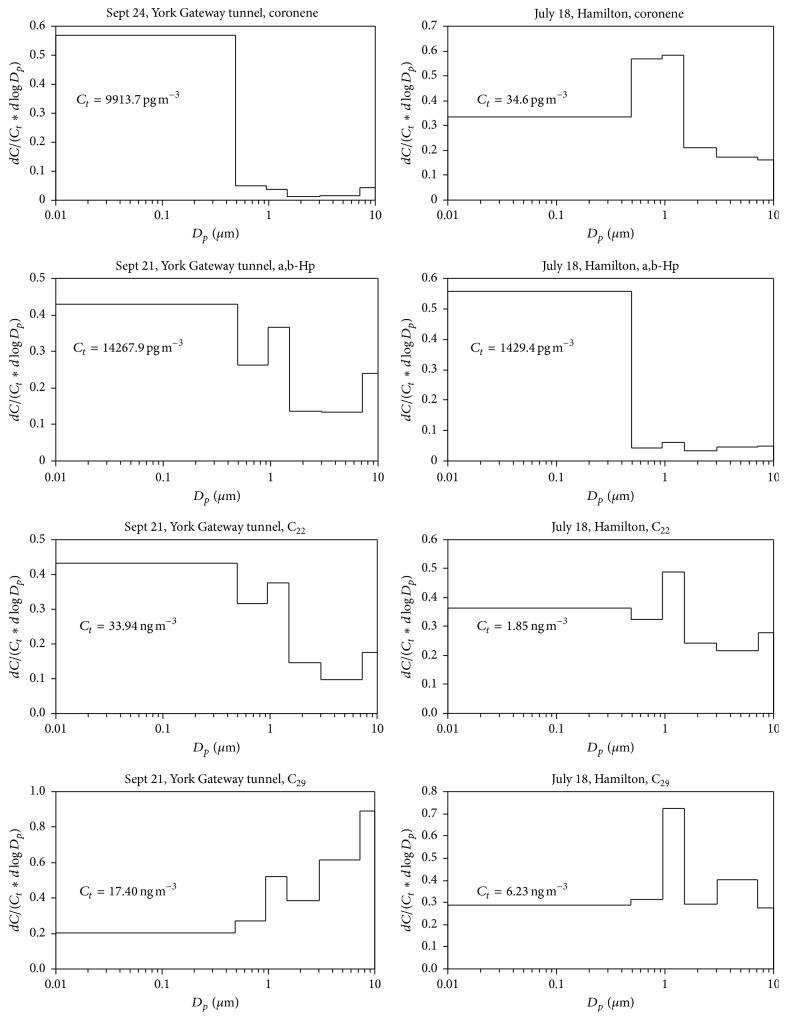
Size distribution of selected molecular marker fractions as a function of size-bin. *C*_*t*_ = the sum of concentrations in all size-bins.

The size distribution of selected molecular marker fractions (*dC*/*C*_*t*_, where *C*_*t*_ is the sum of the marker concentrations in all size-bins) observed at the York Gateway tunnel showed that the majority of Cor, abHp, and C_22_ *n*-alkane were in the size range smaller than 0.4 *μ*m, while the majority of C_29_ *n*-alkane was in the range larger than 0.4 *μ*m (Figure 5). The observations strongly suggest that the origin of Cor, abHp, and C_22_ *n*-alkane was vehicular emissions, while the origin of C_29_ *n*-alkane was road dust, tire debris, or plant wax from the outside of the tunnel. The observations of these makers at Hamilton showed more complex pattern: abHp at Hamilton showed the major distribution smaller than 0.4 *μ*m, similar to that at the York Gateway tunnel, while Cor and C_22_ and C_29_ *n*-alkanes showed another mode around 1 *μ*m. The different size distribution at Hamilton may suggest input of these markers from other sources or particle growth of fine PM emitted from vehicular emissions. More detailed analysis will be needed to explain the observations.

## 4. Conclusion

The simple method with sensitivity correction for determination of nonpolar molecular marker concentrations was established. The procedure requires no preseparation procedure, but routine injection of standard solution to make sensitivity profiles for corrections on variable sensitivity. Standard-spiked test showed the recovery yields ranging from 33 ± 7% to 133 ± 10%. The PAHs were prone to be low recovery yields (33–64%), while the* n*-alkanes were prone to exceed 100% as the carbon number was higher than C_26_. The low recovery yields of the PAHs are probably attributed to their loss during the procedure of extract preparation because of their weak polarity. The significant biases of* n*-alkanes indicate that the biases are likely caused by application of the C_24_ *d*_50_ internal standard to the heavy* n*-alkanes that had significantly different retention time from that of the C_24_ *d*_50_. Sensitivity corrected molecular marker concentrations showed the measured mixing ratios of BaP, Ind, and BgP agreed within the uncertainty of 30% approximately with the reference values of SRM 1650. Analysis of PM_10_ and 6-stage size-segregated filter samples collected from the ambient air showed convincing evidence that the molecular markers can be used to identify and quantify emission sources.

## Figures and Tables

**Table 1:** Selected ions measured by SIM mode.

Time (min)	Selected *m*/*z*
0.0–18.0	Solvent delay
18.0–23.0	85^a^, 99^a^, 152^b^, 178^b^
23.0–29.0	85^a^, 99^a^, 101^b^, 202^b^
29.0–35.0	66^c^, 82^c^, 85^a^, 99^a^, 114^b^, 228^b^
35.0–36.0	85^a^, 99^a^, 126^d^, 252^d^
36.0–37.2	85^a^, 99^a^, 217^b^, 282^b^, 254^e^, 255^e^, 357^b^, 372^b^
37.2–40.4	66^c^, 82^c^, 85^a^, 99^a^, 149^f^, 177^f^, 191^f^
40.4–46.0	85^a^, 99^a^, 138^g^, 139^h^, 149^f^, 177^f^, 191^f^, 276^g^, 278^h^
46.0–51.1	85^a^, 99^a^, 150^i^, 300^i^

^a^Fragment ions of *n*-alkanes. ^b^Molecular and fragment ions from standard chemicals that were not targeted but were contained in the standard mixture. ^c^Fragment ions of C_24_ *d*_50_ and C_30_ *d*_62_ internal standards. ^d^M^+^ and M^2+^ ions of benzo[*a*]pyrene. ^e^M^+^ and its isotopic ions of benzo[*a*]pyrene-*d*_2_ internal standard. ^f^Fragment ions of trisnorhopane, norneohopane, *α*,*β*-hopane, and *β*,*α*-hopane. ^g^M^+^ and M^2+^ ions of benzo[*ghi*]perylene and indeno[*1,2,3-cd*]pyrene. ^h^M^+^ and M^2+^ ions of dibenz[*a*, *h*]anthracene. ^i^M^+^ and M^2+^ ions of coronene.

**Table 2:** List of detection limit (DL) and field blank.

	Mass used for quantitation	Instrumental DL	Field blank	Atmospheric DL
	*m*/*z*	pg	pg m^−3^	pg m^−3^
BaP	255	0.61	0.66	2.3
Db	278	0.71	0.22	0.6
Ind	276	0.43	0.63	3.9
BgP	276	0.32	1.3	4.1
Cor	300	0.37	0.73	2.0

TrisHp	191	1.8	3.6	4.5
NorHp	191	1.5	2.1	4.4
abHp	191	2.3	18	38
baHp	191	1.6	2.6	3.4

C_20_	85	6.9	180	60
C_21_	85	3.3	62	44
C_22_	85	3.2	120	32
C_23_	85	2.6	170	57
C_24_	85	3.2	170	210
C_25_	85	2.6	140	120
C_26_	85	1.9	210	88
C_27_	85	3.1	300	160
C_28_	85	3.9	220	210
C_29_	85	7.0	350	220
C_30_	85	4.4	350	300
C_31_	85	6.4	250	270
C_32_	85	3.9	140	140
C_33_	85	13	110	100
C_34_	85	23	100	92

**Table 3:** Results of standard-spiked test.

	Spiked mass	Determined mass^a^	Offset^b^	Recovery yield^c^	Recovery ratio^d^
	(ng)	(ng)	(ng)	(%)	
BaP*d*_2_	40.0	n.a.^e^	n.a.^e^	33 ± 7	n.a.^e^
Cor	159.6	206 ± 15	46 ± 15	64 ± 22	1.91 ± 0.30
BgP	140.3	150 ± 3	10 ± 3	50 ± 13	1.50 ± 0.08
Ind	140.6	129 ± 2	14 ± 2	43 ± 12	1.29 ± 0.11
Db	140.6	140 ± 4	0 ± 4	46 ± 14	1.38 ± 0.14
BaP	140.1	134 ± 10	−6 ± 10	44 ± 10	1.33 ± 0.09

TrisHp	137.4	121 ± 9	16 ± 9	74 ± 11	1.24 ± 0.04
NorHp	39.2	44 ± 4	4 ± 4	90 ± 17	1.51 ± 0.10
abHp	137.4	135 ± 11	−2 ± 11	83 ± 14	1.39 ± 0.05
baHp	39.2	36 ± 3	−3 ± 3	78 ± 13	1.31 ± 0.06

C_24_ *d*_50_	200.0	n.a.^e^	n.a.^e^	60 ± 8	n.a.^e^
C_20_	198.8	185 ± 20	−14 ± 20	78 ± 12	1.31 ± 0.14
C_21_	151.2	149 ± 16	−2 ± 16	83 ± 13	1.40 ± 0.07
C_22_	274.4	245 ± 22	−30 ± 22	75 ± 10	1.26 ± 0.06
C_23_	168.0	173 ± 15	5 ± 15	87 ± 11	1.46 ± 0.02
C_24_	176.4	178 ± 14	1 ± 14	84 ± 12	1.42 ± 0.03
C_25_	123.2	133 ± 14	10 ± 14	92 ± 13	1.54 ± 0.02
C_26_	154.0	162 ± 15	8 ± 15	88 ± 13	1.48 ± 0.04
C_27_	123.2	170 ± 21	46 ± 21	118 ± 20	1.97 ± 0.10
C_28_	198.8	206 ± 17	7 ± 17	87 ± 14	1.45 ± 0.05
C_29_	135.8	193 ± 13	57 ± 13	117 ± 18	1.96 ± 0.05
C_30_	142.8	207 ± 18	64 ± 18	117 ± 20	1.96 ± 0.10
C_31_	168.0	190 ± 14	48 ± 14	97 ± 13	1.63 ± 0.03
C_32_	145.6	186 ± 13	40 ± 13	107 ± 13	1.79 ± 0.02
C_33_	137.2	198 ± 15	60 ± 13	123 ± 13	2.06 ± 0.06
C_34_	137.2	186 ± 27	48 ± 13	138 ± 10	2.33 ± 0.14

^a^The mass determined by the internal standard method. ^b^Offset = determined mass – spiked mass. ^c^Recovery yields calculated using the C_30_ *d*_62_ ISRC. ^d^The ratio of recovery yield of the substance to the recovery yield of the internal standard (BaP*d*_2_ for the PAHs and C_24_ *d*_50_ for the rest of the compounds). ^e^Not applicable.

**Table 4:** Results of measurement test with SRM 1975 diesel particulate matter extract.

Experiment number	BgP	Ind
	(ng mL^−1^)	
Reference value	50 ± 8	160 ± 13

Exp 1	70	263
Exp 2	52	202
Exp 3	48	190
Exp 4	42	168
Exp 5	41	163
Exp 6	50	184
Exp 7	45	163
Exp 8	47	155
Exp 9	45	142
Exp 10	43	149
Exp 11	58	219
Exp 12	54	195
Exp 13	47	190
Exp 14	52	180

Average	50	183
SD	8	32

**Table 5:** Results of measurement test with SRM 1650 diesel particulate matter.

	BgP	Ind	BaP	Db
	ppm			
1st reference values^a^	2.4 ± 0.6	3.2 ± 0.5	1.2 ± 0.3	n.a.^d^
2nd reference values^b^	6.5 ± 0.98	5.6 ± 0.53	1.3 ± 0.36	n.a.^d^
3rd reference values^c^	6.04 ± 0.30	4.48 ± 0.12	1.25 ± 0.12	0.365 ± 0.082

Exp 1	5.3	4.0	1.4	0.87
Exp 2	4.8	3.6	1.3	0.59
Exp 3	3.3	2.5	0.91	0.48

Average	4.47	3.37	1.20	0.65
SD	1.04	0.78	0.26	0.20
The error of the mean	0.60	0.45	0.15	0.12

^a^SRM 1650 (1985). ^b^SRM 1650a (2000). ^c^SRM 1650b (2013). ^d^The reference value was not given.
